# Supplementary material for: Storms drive outgassing of CO2 in the subpolar Southern Ocean
Source: Nat Commun. 2022 Jan 10;13:158. doi: 10.1038/s41467-021-27780-w (PMC8748750; doi:10.1038/s41467-021-27780-w)
Supplement: Supplementary file 1 — Supplementary Information [file 41467_2021_27780_MOESM1_ESM.pdf]

Supplementary Materials for

Storms drive outgassing of CO_2 in the subpolar Southern Ocean

S-A Nicholson[1*], D. B Whitt[2,3], I. Fer[4], M.D. du Plessis[1,5,6], A.D Lebéhot[1,6,7], S. Swart[5,6], A.J Sutton[8], P.M.S Monteiro[1,6]

*Corresponding author. Email: snicholson@csir.co.za

This PDF file includes:

Supplementary figures S1 to S9

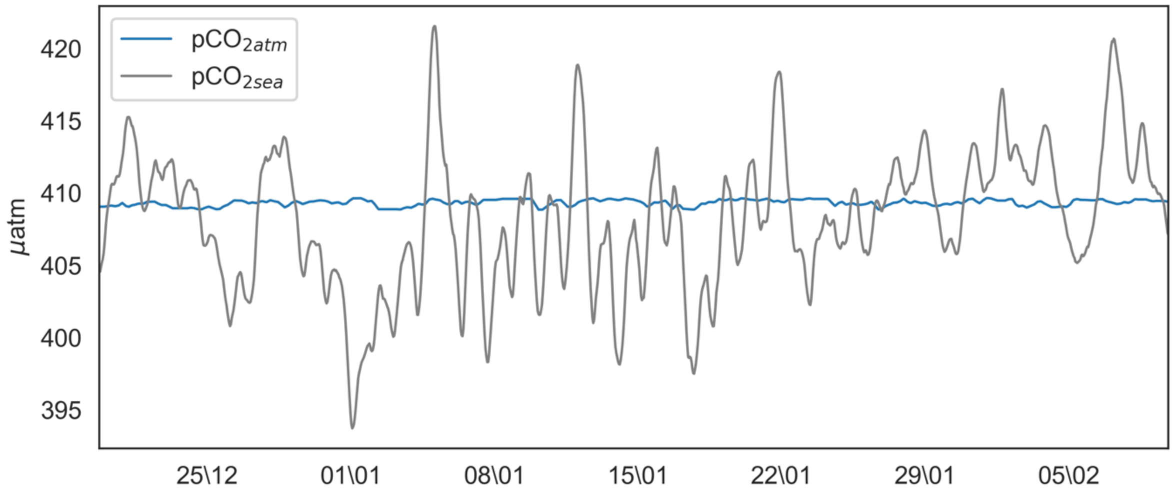

Supplementary Figure 1.

Wave Glider atmospheric partial pressure of CO_2 (pCO_{2atm}) and the oceanic partial pressure of CO_2 (pCO_{2sea}) in μatm measured during summer Dec 2018 - Feb 2019. Time is given as dd\mm.

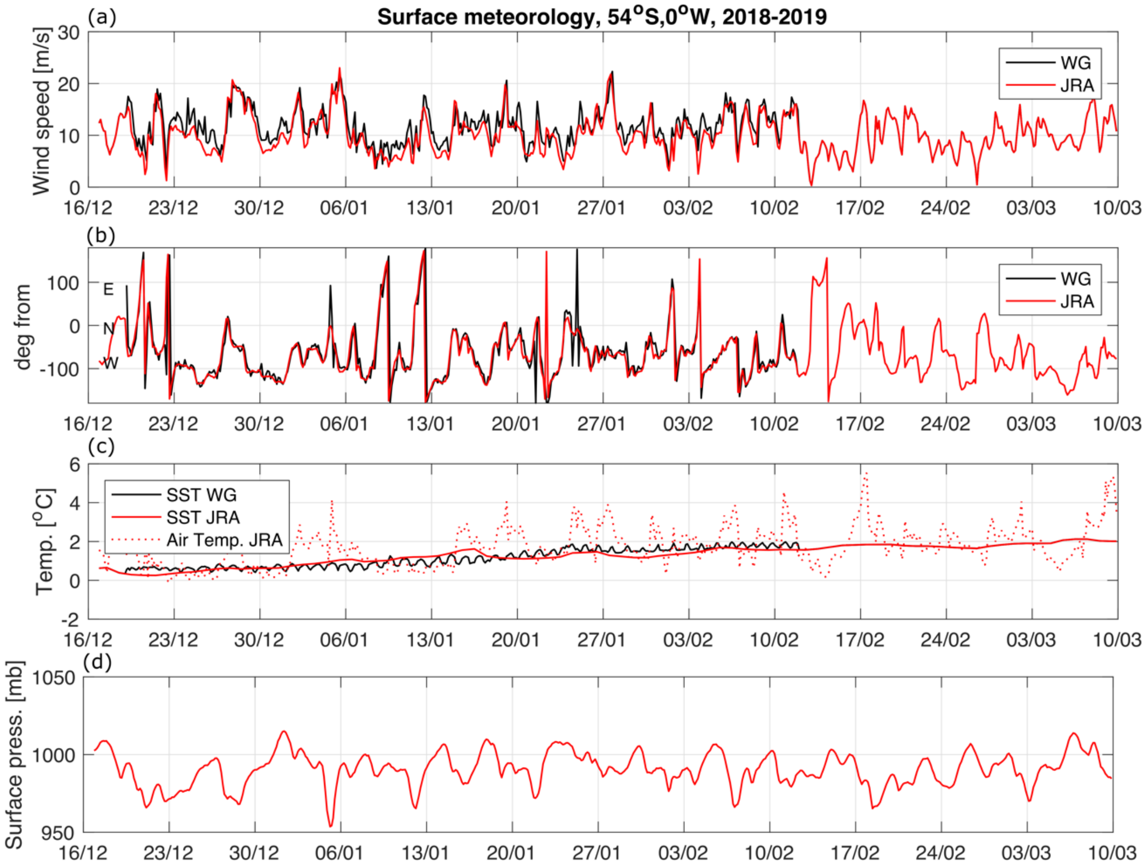

Supplementary Figure 2.

Meteorological data from Wave Glider (black) compared with collocated reanalysis product JRA-55-do (red)[68]. (a) Wind Speed (m s^{-1}), (b) Wind direction (˚), (c) sea surface temperature (SST, ˚C), (d) Sea level pressure [mb]. Time is given as dd\mm of 2018 and 2019.

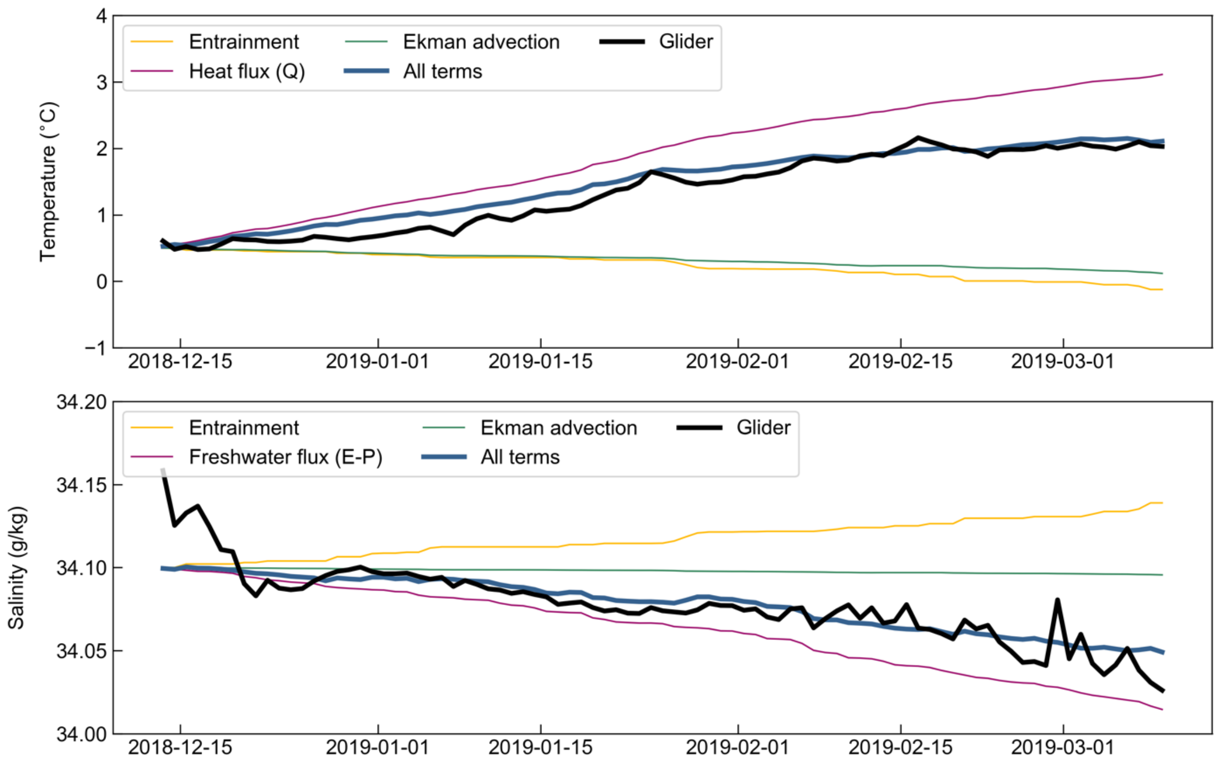

Supplementary Figure 3.

Evolution of mixed layer temperature (a) and salinity (b) as observed from the Slocum glider (black line) and estimated from the mixed layer budget equations for air-sea flux heat and freshwater fluxes (purple line), Ekman advection (green line) and entrainment (yellow line). The sum of all the budget terms is shown as the blue line.

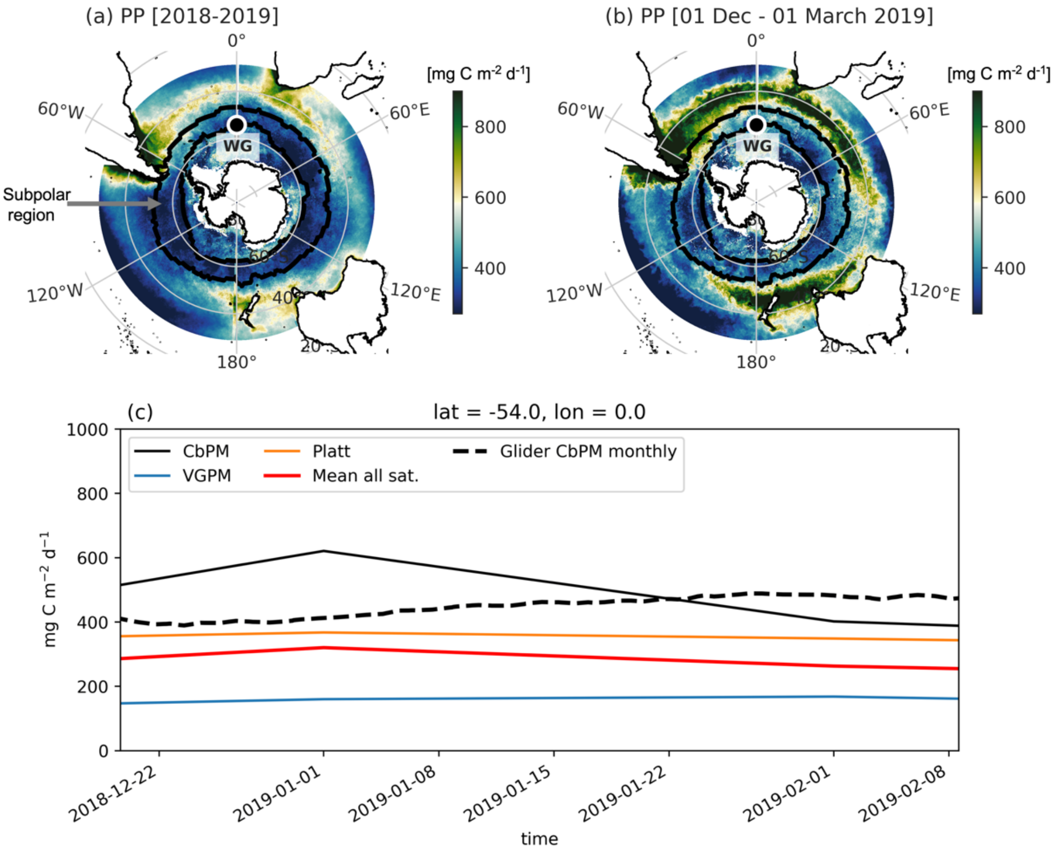

Supplementary Figure 4.

Monthly mean net primary productivity (NPP, in units mg C m^{-2} d^{-1}) derived from satellite ocean color using three different primary models: the Carbon-based Productivity Model (CbPM[72]), the Vertically Generalized Production Model (VGPM[73]) and the Platt model[74]. The mean of the three primary production models is shown in (a) averaged for 2018-2019 and (b) averaged over summer 2018-2019 coinciding with the deployment of the Gliders. (c) The time-series of satellite derived NPP using the three primary production model estimates are compared with the estimate derived by applying the Carbon-based Productivity Model[72] to optical measurements on the Slocum glider (dashed-line).

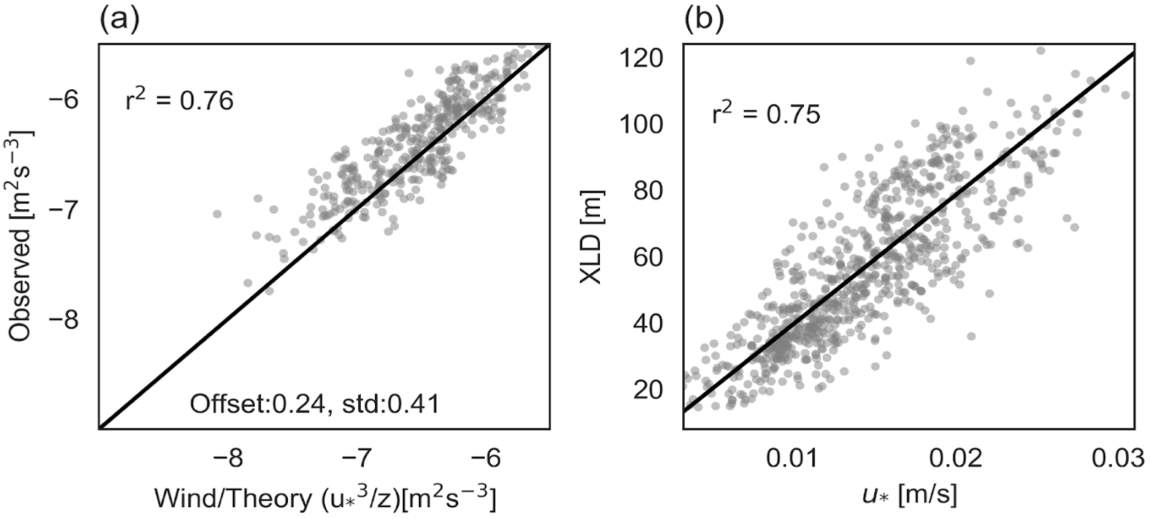

Supplementary Figure 5.
(a) Relationship between theoretical (u_*^3/z) dissipation rates and glider-observed dissipation rates averaged over 6-15 m depth (on log10 scales) over the two months of the field experiment. (b) Relationship between the mixing layer depth (XLD, defined as in [38]) and u_*. A rolling mean of window size T/4, where T is the inertial period (i.e., about 4 hours) has been applied to both (a) and (b).

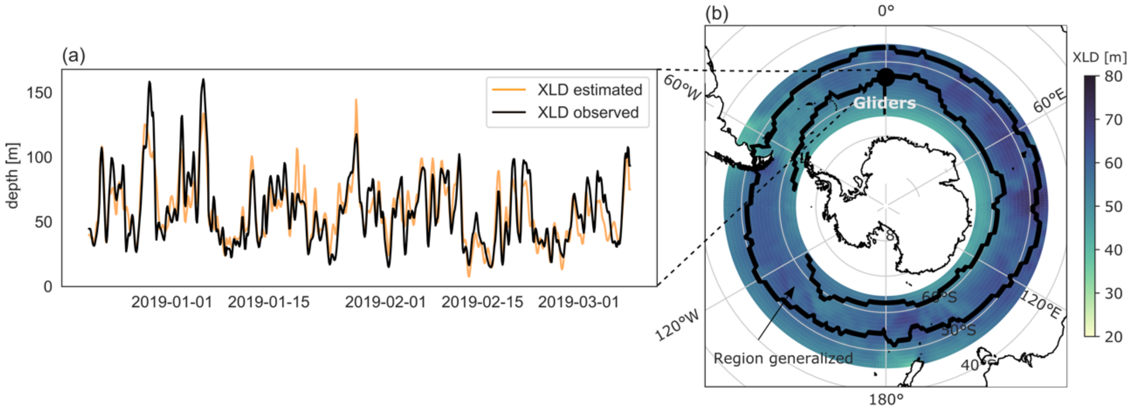

Supplementary Figure 6.
Generalisation of the mixing layer depth (XLD) across the subpolar Southern Ocean. (a) XLD observed (m) vs estimated (m) based on the relationship found in Fig. S5. (b) The mean summer XLD (m) estimated for the entire Subpolar region between 2015 - 2019, black contours mark the climatological ice edge maximum [34] and CO_2 outgassing maximum as defined in Fig 1a, computed using CSIR-ML6 data[1,33].

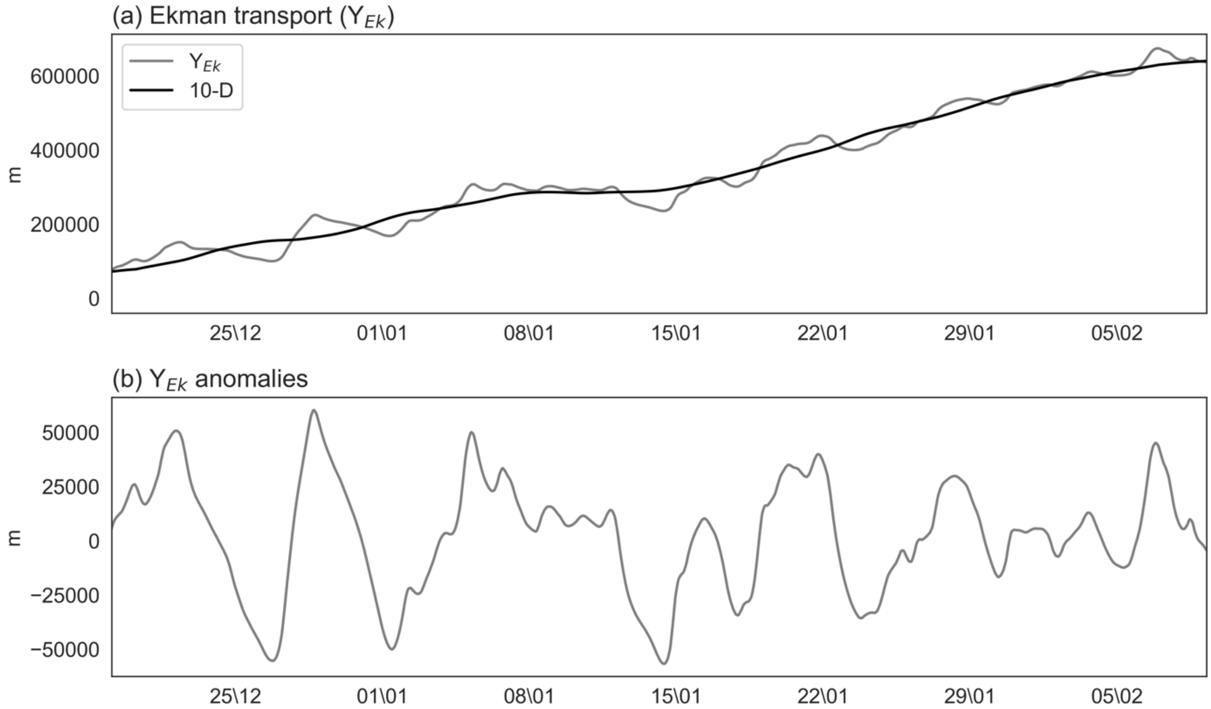

Supplementary Figure 7.
The cumulative daily Ekman transport Y_{Ek} (grey) in meters (m) with the 10-day rolling mean shown in black. (b) the corresponding synoptic Y_{Ek} anomalies (m) computed by removing the 10-day mean from Y_{Ek} in (a).

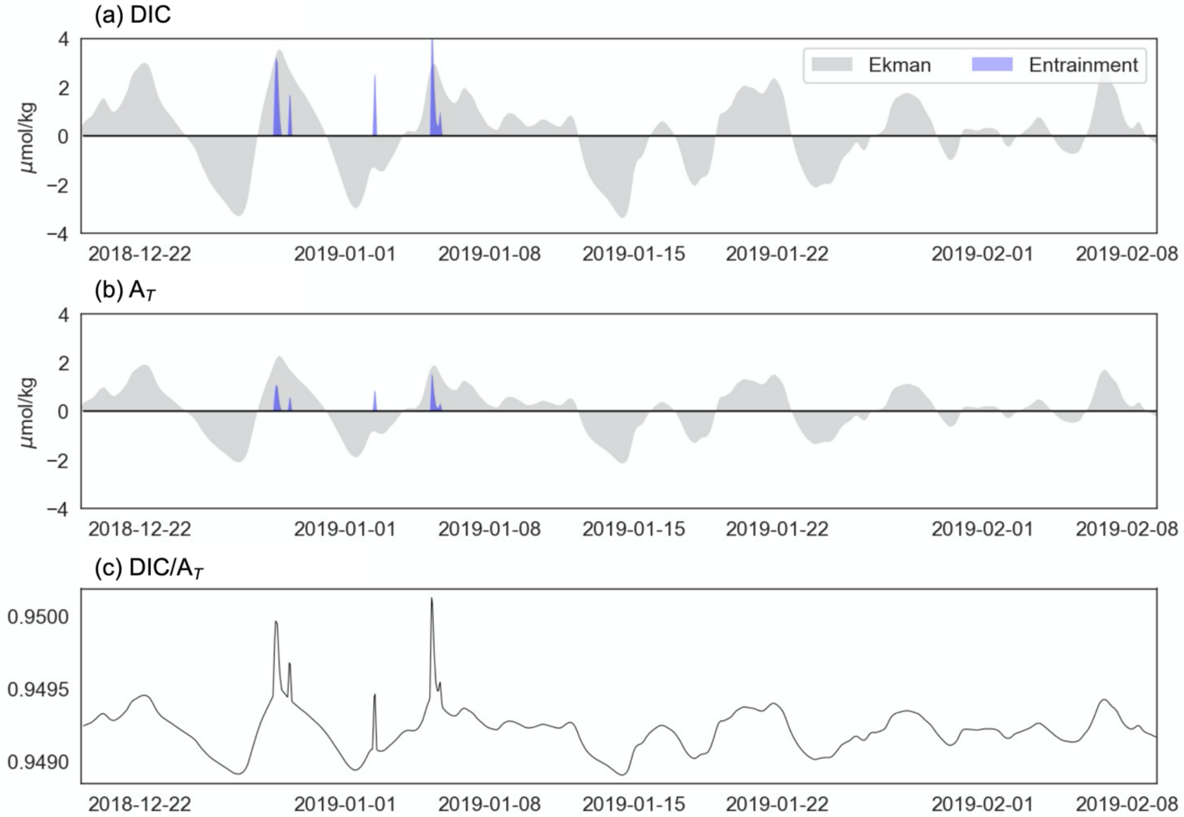

Supplementary Figure 8.
The modelled Ekman (grey) and entrainment (blue) driven anomalies for (a) dissolved inorganic carbon (DIC, μmol kg^{-1}) and (b) total alkalinity (A_T, μmol kg^{-1}) at the location of the gliders calculated following Eq. 2-4. These are used to estimate the synoptic anomalies in $pCO_{2\text{-DIC}}$' show in in Fig 3d. (c) The corresponding ratios of DIC/A_T due to Ekman + Entrainment.

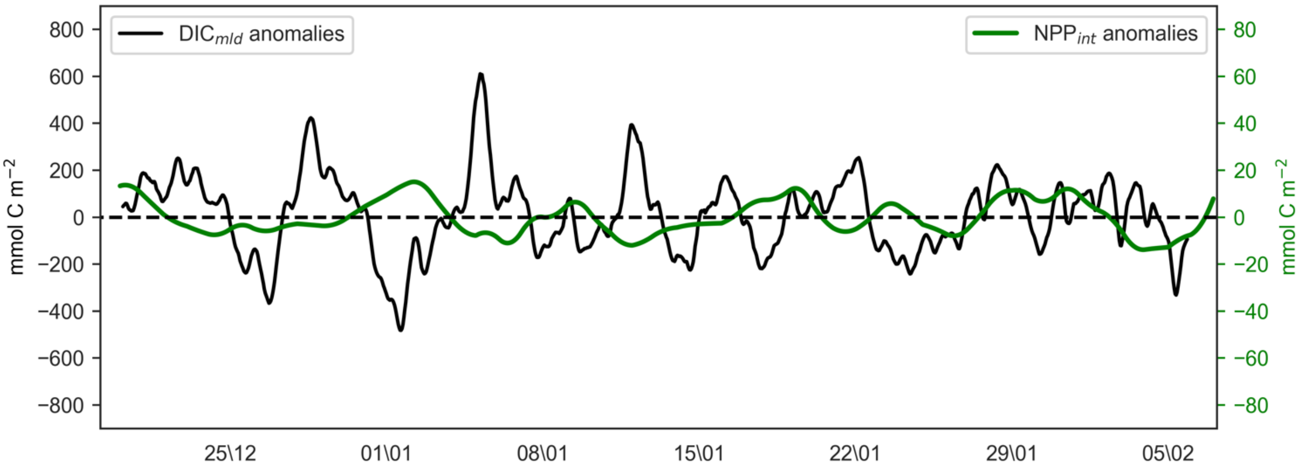

Supplementary Figure 9.
Synoptic anomalies (the residual after removing a 10-day rolling mean) of dissolved inorganic carbon (DIC) scaled by the mixed-layer depth (DIC_{MLD} in units mmol C m^{-2}, left axis), where DIC_{MLD} = DIC*MLD. The MLD is estimated from the profiling glider. DIC is estimated from Wave Glider observed pCO_2 and derived Total Alkalinity[66] using PyCO2SYS[67]. DIC_{MLD} is compared to the synoptic anomalies in time integrated net primary production (NPP_{INT} in units mmol C m^{-2}, right axis; note that the right axis is an order of magnitude smaller than the left axis) derived from the glider as described in Fig. S4.
